# Praxis makes perfect?

**DOI:** 10.1192/bjb.2018.106

**Published:** 2019-01-22

**Authors:** Cate Bailey

**Affiliations:** East London NHS Foundation Trust

**Keywords:** Education and training, information technologies, stigma and discrimination

## Abstract

Cate Bailey outlines the origin and goals of the new trainees' section of the *BJPsych Bulletin*. ‘Praxis’ will involve two components. The first will be clinically inspired and problem based. Trainees will have an opportunity to collaborate with experts from different disciplines to examine how existing literature and knowledge can be applied to practice, with a clinical scenario forming the basis for discussion. The second component will be made up of commissioned or proposed editorial-type articles on subjects which fall broadly under the heading of personal and professional development. This will offer a space for reflection on relevant contemporary topics such as resilience, burnout, supervision, and patient involvement and co-production.

**Declaration of interest:**

C.B. is editor of the *BJPsych Bulletin* trainees' section.

It was a surprising, not to mention slightly terrifying honour to be offered the role of Editor of the new trainees' section of the *BJPsych Bulletin*. Three years after achieving my membership of the Royal College of Psychiatrists, I find I have stumbled into the heart of the institution itself. So perhaps I am justified in experiencing an episode of acute (on chronic) imposter syndrome. Each of the RCPsych journals were, to my colleagues and I training in Australia, the most esteemed publications one could imagine. Until now, my own attempts to grace these pages had been limited to poetry – though those two small references, I think, have added significant gravitas to an otherwise relatively slim academic CV.

The *BJPsych Bulletin* has a long history of offering trainees opportunities where they can gain editing experience. Since 1999, the *Psychiatric Bulletin*, as it was formerly known, held posts for trainees to sit on the Editorial board, engage in peer reviewing and develop an individually selected project during their three-year tenure.^1^ This new post, however, represents a unique challenge to create an entirely new section focusing on trainees and education. It is already apparent that the *Bulletin* regularly publishes on topics of education and training, and on clinical issues relevant to trainees. This is perhaps why it is one of the most frequently read journals among trainee psychiatrists in the UK.^2^ So, how to take this further?

While preparing for the interview for the post, I allowed my mind to run wild with all the things I would like to read about: issues I felt were pertinent to the trainees of today, and the psychiatrists of tomorrow, in the current climate of uncertainty and austerity. These are strange times indeed. Core trainee recruitment is up by a third, at least in part thanks to the RCPsych #choosepsychiatry campaign, which aimed to challenge the perceptions that patients with mental health conditions don't get better, and portrayed the specialty as a unique chance to understand a person from multiple perspectives.^3^ These figures are more impressive still, given that applications to specialty training are falling overall and junior doctors are apparently leaving in droves.^4^ However, the life of psychiatry trainees is far from rosy, as eloquently outlined in the trainee-led review into morale and training in psychiatry, aptly titled ‘Supported and Valued?’.^5^

The new section, is of course, a work in progress and will no doubt evolve in response to the limits of reality, time and what is achievable in the medium of print. Having said that, one of my goals is to find ways to engage readers beyond the traditional journal form, through online content, Twitter discussions and podcasts. Although a reluctant millennial, and generally inept user of social media, I've been inspired in this area by my enthusiastic geriatrician colleagues @MDT_podcast, and by psychiatrists Katherine Adlington (clinical editor @bmj_latest and @Kateadlington), Derek Tracy (@Derektracy1; Editorial board member and social media leader @TheBJPsych) and none other than the Twitter queen herself: our distinguished president Wendy Burn (@wendyburn). These represent a handful of the growing number of clinicians and researchers who are connecting creatively, finding novel and inclusive ways of sharing, discussing and debating information. The Bulletin will be regularly tweeting updates including news about the trainees' section via #BJPBulletin.

Naturally the first task when developing any new section (after buying fresh stationery) is to think of a snappy name. Inspired by phenomenology and psychopathology, I found myself immediately searching for German words. It was tempting to settle upon ‘weltschmerz’ (meaning world weary, or ‘mental depression or apathy caused by comparison of the actual state of the world with an ideal state’).^6^ Though perhaps a little too sullen, it did capture something of the zeitgeist and the mood of many junior doctors. Other contenders, although clever, were slightly esoteric, e.g. ‘verstehen’ (‘empathic understanding of human behaviour’).^7^ Perhaps if I had been more Twitter-savvy I would have conducted a poll. But the aforementioned limits of reality (particularly time) meant it had to be an executive decision. And so we came upon ‘Praxis’.

Derived from Greek, praxis means ‘the exercise or practice of an art, science, or skill’ or ‘practical application of a theory’.^6^ In a more occupational or neurological sense, it is the ability to plan or execute movement (https://medical-dictionary.thefreedictionary.com/). It also has further connotations in educational theory. Paulo Freire, one of the most influential thinkers about education in the late 20th century, regarded praxis as more than an action based on prior knowledge. Rather, he described a dedication to human well-being, truth, and a perpetually shifting interaction between question and answer: the application of knowledge to action for change.^8^ That sounds a lot like psychiatry, and what I hope we can achieve in some small way with this section.

The section will involve two components. The first has been modelled on *BMJ* Endgames, and is clinically inspired and problem based. This section aims to capture the fertile discussion of local case presentations with more comprehensive analysis and varied perspectives. Psychiatric presentations sit at the intersection of the biomedical, social, cultural, and interpersonal, the past and the present. This section will explore these often knotty and interconnected aspects, with a clinical scenario forming the framework for discussion.

Trainees will have an opportunity to collaborate with experts from different disciplines to examine how existing literature and knowledge can be applied to practice. In any given scenario, possible avenues for discussion could include neuroscience, diagnosis, formulation, treatment, transference and countertransference, practicalities, real-world service provision, and ethical and legal considerations.

The College is in the process of developing a new curriculum, and the collaboration with the Gatsby Foundation and the Wellcome Trust has already seen ‘Brain Camps’ being held around the country.^9^ This new section may be a place where new neuroscience can be woven into a holistic approach. Patient involvement will be strongly encouraged, and we have been helpfully guided by the *BMJ* in this regard.^10^ A template for these clinical types of paper will be available on the website shortly.

The second component will be made up of commissioned or proposed editorial-type articles on subjects which fall broadly under the heading of personal and professional development. This will offer a space for reflection on relevant contemporary topics. Subjects I hope we can explore include psychodynamic and systemic approaches to resilience and burnout, consideration of what supervision should entail, trainee experiences of organising their own personal therapy, the experience of discrimination in psychiatric training, and what meaningful patient involvement and co-production looks like in practice and education. In commissioning for this section, I propose to work closely with the Psychiatry Trainees' Committee and the College to ensure we are responding to the needs of trainees.

As Dr Poole has said in his editorial, the future of the journal depends on you.^11^ The success of the trainees' section depends on trainees and trainers pitching ideas for articles. If you're facing a complex clinical scenario, the chances are that someone else is too. And if you think you might never see your name in this journal – believe me, I've been in your shoes. Praxis is about dialogue, so get in touch. As Freire said, ‘knowledge emerges only through invention and reinvention, the restless, impatient, continuing, hopeful inquiry beings pursue with the world and with others’.^12^

## About the author

**Cate Bailey**, ST6 in Old Age and General Adult Psychiatry, Isle of Dogs Community Mental Health Team, East London NHS Foundation Trust, UK.
